# Trend in major neonatal and maternal morbidities accompanying the rise in the cesarean delivery rate

**DOI:** 10.1038/srep12565

**Published:** 2015-07-29

**Authors:** Sivan Zuarez-Easton, Eliezer Shalev, Raed Salim

**Affiliations:** 1 Department of Obstetrics and Gynecology, Emek Medical Center, Afula, Israel; 2 Rappaport Faculty of Medicine, Technion, Haifa, Israel

## Abstract

The aim of the study was to explore a cesarean delivery rate (CDR) beyond which major neonatal and maternal morbidities may outweigh the benefits of the procedure itself. A retrospective population-based cohort study was conducted at a single university teaching hospital between 1993 and 2012. Pregnant women who delivered at a gestational age of 23 weeks or more were included. Data including delivery mode, brachial plexus injury (BPI), neonatal encephalopathy (NE), placenta accreta (PA), blood transfusion (BT), and cesarean hysterectomy (CH) for each year were extracted, plotted, and trends analyzed. The Cochran-Armitage Trend Test was used to identify trends and correlations. Overall, 83,806 deliveries took place during this period. CDR increased from 10.9% to 21.7% (*p* < 0.001). Significant decreases in the incidence of BPI (*p* < 0.001) and NE (*p* = 0.006) were observed. At CDRs of 13.6% and 20%, there was no further significant decrease in the incidence of BPI and NE, respectively. The incidence of BT increased significantly (*p* < 0.001) while the increase in the incidence of PA was not significant (*p* = 0.06) nor the change in the incidence of CH (*p* = 0.4). A CDR of 20% may still confirm additional beneficial effect on major perinatal morbidities without a significant increase in the incidence of PA.

Cesarean delivery is a major obstetrical surgical procedure that aims to save the lives of mothers and fetuses^1^. Over the last decades, the incidence of cesarean deliveries both primary and repeated has risen dramatically^2^. This rise also has to do with the rising maternal age, the reduction of number of children per family in the so called “first world”, and maternal request for cesarean delivery. Nevertheless, the impact of this dramatic rise on neonatal morbidity and mortality, or maternal health is still challenged^3^. Several studies have documented an increased incidence of placenta previa and placenta accreta as a result, and an incidence of 1% of hysterectomy among women who undergo multiple cesarean deliveries most likely as a result of abnormal placentation^3^,^4^. While cesarean delivery is designated to prevent or treat life-threatening maternal or perinatal complications^5^, an appropriate rate of use should be one associated with the lowest reasonable level of maternal and perinatal morbidity and mortality.

The appropriate overall rate of cesarean delivery is not easily determined as it varies according to multiple factors and populations. The most known recommended figure is the 15% upper limit suggested by the World Health Organization (WHO) in 1985^6^, with some evidence indicating that rates above 15% are not associated with additional reduction in maternal or neonatal mortality and morbidity^7^.

Appropriate cesarean delivery rates should be determined through an outcome-based approach. A properly powered observational study that would provide such data requires thousands of women, given the relatively low incidence of major perinatal and maternal morbidity and mortality that are related to the mode of delivery.

In this study we aimed to determine the trend of overall cesarean delivery rate at a single university teaching hospital during the last two decades and to explore a cesarean delivery rate beyond which major neonatal and maternal morbidities may outweigh the benefits of the procedure itself.

## Results

Overall 83,806 deliveries took place during the study period and all were included.

Demographic and obstetric characteristics of women included are presented in Table 1. An increase in the incidence of diabetes was observed during the study period (*p* < 0.001). Other demographic variables did not change significantly over time.

**Figure 1:**
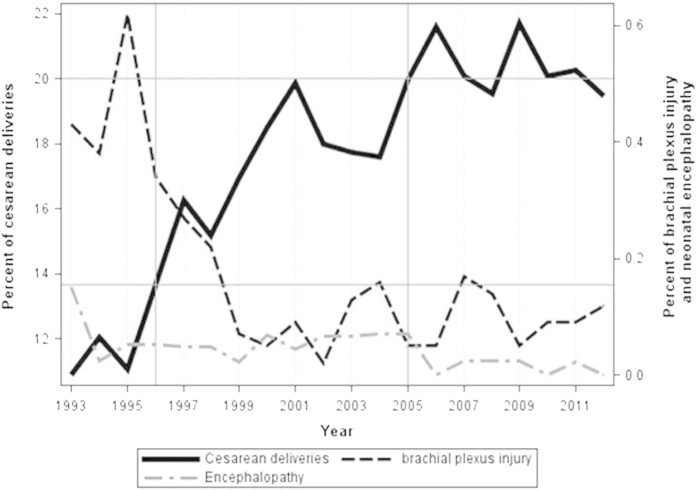
Trends in major neonatal morbidities rates and in cesarean delivery rate 1993–2012 .

During the study period, the overall cesarean delivery rate increased from 10.9% in 1993 to a maximum rate of 21.7% (*p* < 0.001) as seen in Table 2. Additionally the trend of both elective and non-elective cesarean deliveries changed significantly (*p* < 0.001) during the study period as seen in this Table. At the same time, the incidence of brachial plexus injury dropped significantly (*p* < 0.001) from 4.3 cases per 1000 deliveries in 1993 to 1 per 1000 deliveries within the last 3 years (Table 2 and Fig. 1). At a cesarean delivery rate of 13.6% there was no further significant decrease in the incidence of brachial plexus injury (Fig. 1). Below this cutoff, the incidence of brachial plexus injury was 3.76 times greater compared to the period with a cesarean delivery rate above this cutoff (OR 3.76; 95% CI 2.71–5.16).

The incidence of neonatal encephalopathy dropped significantly (*p* = 0.006) from 1.5 cases in 1993 to less than 0.2 per 1000 deliveries within the last 3 years (Table 2 and Fig. 1). There was no further significant decrease in the incidence of neonatal encephalopathy at a cesarean delivery rate of 20% or more (Fig. 1).

**Figure 2:**
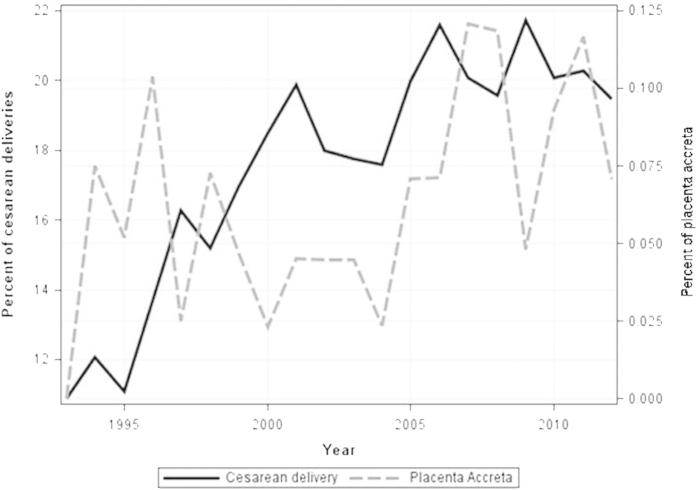
Trends in placenta accreta rate and in cesarean delivery rate 1993–2012 .

**Figure 3:**
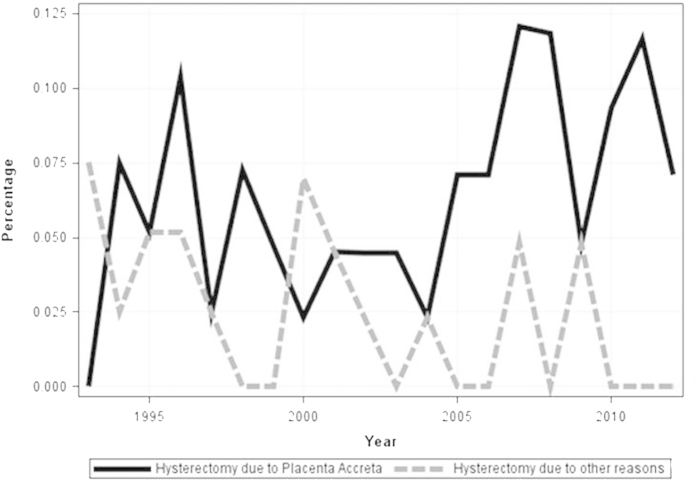
Trends in cesarean hysterectomy rate due to placenta accreta and due to all other reasons beside placenta accreta 1993–2012 .

**Figure 4:**
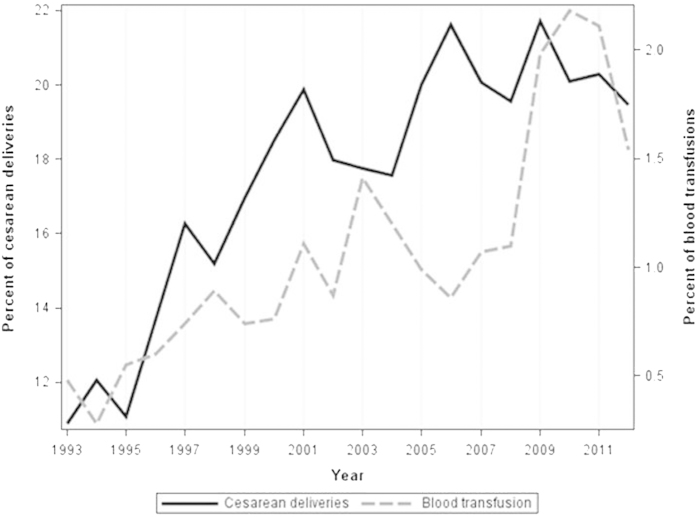
Trends in blood transfusion rate and in cesarean delivery rate 1993–2012.

The increase in the cesarean delivery rate was accompanied with an increase in the incidence of placenta accreta, although the trend up to a maximum cesarean delivery rate of 21.7% was not significant (*p* = 0.06) (Fig. 2). There was no significant change in the incidence of cesarean hysterectomy (*p* = 0.4). Although the increase in the cesarean hysterectomy rate performed due to placenta accreta was not significant (*p* = 0.06), there was a significant decrease in the rate of hysterectomies performed due to other reasons (*p* = 0.02) (Fig. 3). Additionally, a significant increase in the incidence of blood transfusion was observed during the study period (*p* < 0.001). This trend in the blood transfusion rate was sustained as the cesarean delivery rate increased (Fig. 4).

Multivariate logistic regression analysis was used to estimate the odds for brachial plexus injury during the study period adjusting for maternal age, diabetes, mode of delivery, year of birth, number of fetuses, and maternal country of birth. For every additional year, from 1993 to 2012, a mean reduction of 12% in the incidence of brachial plexus injury was observed (OR 0.89; 95% CI 0.87–0.92). Using the same analysis, a mean increase of 6% in maternal composite morbidity was also observed for every additional year, from 1993 to 2012 (OR 1.06; 95% CI 1.05–1.08).

One case of maternal death occurred during the study period. This woman delivered vaginally.

## Discussion

The overall cesarean delivery rate at a single university teaching hospital doubled within the last two decades. At the same time, the incidence of major neonatal morbidities declined by a factor of 3 to 5 times. No further reduction in neonatal morbidities was observed at a cesarean delivery rate of 20% or more. The rate of blood transfusion increased continuously during the study period. The incidence of placenta accreta and cesarean hysterectomies performed due to placenta accreta did not increase significantly.

Caesarean delivery is an invasive intervention performed to prevent or treat life-threatening maternal or perinatal complications^7^. Ananth *et al.* reported that between 1990 and 2004 a temporal increase in preterm cesarean delivery rate was found among preterm gestations in the United States. This increase was associated with improved perinatal survival mainly due to dramatic incremental declines in stillbirths^8^. Landon *et al.* compared maternal and perinatal outcome among women who had a trial of labor after a previous cesarean or elective repeated cesarean delivery. The frequency of hysterectomy and maternal death did not differ between the groups. However, the incidence of encephalopathy was significantly lower among infants whose mothers underwent elective repeated cesarean delivery, although the absolute risk was small (0.46 per 1000)^9^.

The results of the current study provide comparable evidence for the potential benefit in reducing the incidence of major neonatal morbidities. Still, this intervention may have major implications on a woman’s health in the present pregnancy, potential future morbidity in subsequent pregnancies, including abnormal placentation, and significant cost implications for the health care providers^10^. The increased incidence of blood transfusion observed during the study period confirms the recent increased trend observed in many other developed countries^11^,^12^,^13^,^14^,^15^. However, the increase in cesarean delivery rate over time and the increase in the rate of placenta accreta account for only a small fraction of this rise according to several reports^14^,^15^. Additionally, previous reports were unable to explain the temporal trend in severe postpartum hemorrhage despite their inclusion of a large number of potential risk factors^14^,^15^.

The appropriate cesarean delivery rate should be associated with the lowest reasonable maternal and perinatal morbidity and mortality^5^. Nevertheless, in the last decades the cesarean delivery rate has risen remarkably and inappropriately, and cesarean delivery rates of up to 85% have been reported^1^. Assuming that there is an appropriate cesarean delivery rate, it should be based on an outcome-centered approach. A lower limit range from a minimum of 1% to an optimum target of 5% has been recommended to avoid death and severe maternal morbidity^16^,^17^,^18^. These figures are good estimates based on complication rates in the mother^16^,^17^ and on historical data^5^, but it is still unknown whether these rates of intervention are enough to prevent avoidable perinatal morbidities. Two observational studies^5^,^19^ that included developing countries assessed the association between the caesarean delivery rate and mortality and morbidity in mothers and neonates. Both studies recorded no reductions in maternal and neonatal mortality and morbidity when the cesarean delivery rate was more than 15%. Moreover, an increased rate of intervention was associated with higher mortality and morbidity in mothers and neonates^19^. These observations seem to confirm the most recommended figure of 15%, suggested by the WHO in 1985^6^. Nevertheless, both studies investigated maternal and perinatal mortality while major neonatal morbidities that were examined in the current study were not investigated.

Li *et al.* compared perinatal outcomes in high income countries in 1996 and 1997 among obstetric practices with low (less than 18%), medium (18–27%), and high (above 27%) cesarean delivery rates. Perinatal mortality rates were comparable among the three groups. A small but significant increase in intracranial hemorrhage was observed among infants delivered by low-rate physicians (CDR less than 18%) compared with those delivered by medium and high-rate physicians^20^. Althabe *et al.* reported no association between cesarean delivery rates in medium and high income countries and maternal or neonatal mortality. For that reason, the authors called for good quality research to assess the effect of the high figures of cesarean delivery rates on maternal and neonatal morbidity^5^.

Limitations of this study are those inherent in the use of retrospective databases and the use of ICD-9 codes. Prenatal and intrapartum complications may not be accurately recorded. However, inaccuracies in the current study were minimized by use of multiple sources for identifying specific maternal and neonatal outcomes when possible, and by manual validation of individual medical files. Additionally, several factors may influence the cesarean delivery rate and affect the incidence of major maternal and neonatal morbidities. For that reason, the change in the cesarean delivery rate occurring in the last 20 years may not solely explain the changes in the occurrence of major maternal and neonatal morbidities.

The cesarean delivery rate of 15% suggested by the WHO^6^ is considered the best known recommended upper limit^7^. However, this rate is driven from low income countries and may not be applicable in contemporary obstetric practice in high income countries. According to the current study a rate of 20% may still provide additional beneficial effect on major perinatal morbidities without a significant increase in the incidence of placenta accreta.

## Methods

A retrospective population-based cohort study conducted at a single university teaching hospital between January 1993 and December 2012. Data was obtained from the hospital discharge register with the International Classification of Diseases, Ninth Revision, and Clinical Modification codes crosschecked with the labor electronic medical records. Data that was collected included the number of deliveries occurring during the study period, cesarean deliveries, and major neonatal and maternal morbidities. Cesarean deliveries were further stratified to elective and non-elective. Major neonatal morbidities that were considered included brachial plexus injury and neonatal encephalopathy. Major maternal morbidities that were considered included placenta accreta, blood transfusion indicated for peripartum hemorrhage, and cesarean hysterectomy. Reasons for cesarean hysterectomy were then reviewed and categorized as to whether they were performed due to placenta accreta. Obstetricians on duty made the decision on performing a cesarean section. Decision for cesarean hysterectomy at our institution was made after consultation (antepartum or intra-operatively) with one (or more) of the senior obstetricians that are available 24/7. In order to minimize bias, medical files of cases identified with major morbidity were manually checked and validated.

The diagnosis of both brachial plexus injury and neonatal encephalopathy were made before neonatal discharge by the neonatologists. Any upper-arm weakness (paresis) or paralysis was categorized as a brachial plexus injury and included in the analysis. The diagnosis of encephalopathy was based on neurologic exam. The clinical criteria for the diagnosis of encephalopathy included level of consciousness, muscle tone, tendon reflexes, presence of myoclonus and seizures, complex reflexes, and autonomic function^21^. The diagnosis of placenta accreta was made by histology in cases where cesarean hysterectomy was performed or according to the surgeon’s judgment when parts of the placenta were heavily attached and left *in situ*.

Inclusion criteria were all pregnant women who gave birth at our institution at a gestational age of 23 weeks or more.

Using the above database, the rate of cesarean deliveries was obtained for all births per year and the trend in the annual rate was then calculated. The rates of major maternal and neonatal morbidities were also calculated for each year and trends were then determined.

Primary outcome was to explore the association between the trend of cesarean delivery rate during the last two decades and the occurrence of major neonatal morbidities. The study protocol was approved by the local Institutional Review Board.

### Statistical analysis

The statistical analysis was performed using SAS 9.2 software (SAS Institute Inc., Cary, NC, USA). Trend analysis was performed using the Cochran-Armitage Trend Test. Significant trend was considered when *p*-value < 0.05.

Logistic regression analysis was used to estimate the odds for brachial plexus injury during the study period and a maternal composite morbidity that included placenta accreta, blood transfusion, and cesarean hysterectomy. Maternal age, maternal country of birth (Israel versus other countries), diabetes (gestational or pre-gestational versus none), mode of delivery (cesarean versus vaginal delivery), number of fetuses (singleton versus multiple gestation), and year of birth were entered as covariates. Odds ratios and the 95% confidence intervals were calculated.

The sample size during a period of 20 years was sufficient to show a 50% reduction in the incidence of brachial plexus injury (from an expected rate of 0.4 to 0.2%) with a level of significance of 95% (α = 0.05) and a power of 80% (β = 0.2).

## Additional Information

**How to cite this article**: Zuarez-Easton, S. *et al.* Trend in major neonatal and maternal morbidities accompanying the rise in the cesarean delivery rate. *Sci. Rep.* **5**, 12565; doi: 10.1038/srep12565 (2015).

## Figures and Tables

**Table 1:** Demographic and Obstetric Characteristics.

Year	Births	Maternal age	Natives	Diabetes in pregnancy	Multiple gestation*	Male neonates	Neonatal birthweight
1993	3988	29.0 ± 5.8	3162 (79.3)	148 (3.7)	80 (2.0)	2074 (52.0)	3218.6 ± 615.6
1994	4000	29.0 ± 5.7	3256 (81.4)	120 (3.0)	72 (1.8)	2040 (51.0)	3212.5 ± 608.4
1995	3854	28.8 ± 5.7	3191 (82.8)	123 (3.2)	89 (2.3)	1923 (49.8)	3211.7 ± 613.0
1996	3861	28.8 ± 5.7	3193 (82.7)	135 (3.5)	77 (2.0)	1992 (51.6)	3236.4 ± 619.6
1997	4023	28.6 ± 5.6	3407 (84.7)	173 (4.3)	101 (2.5)	2028 (50.4)	3212.3 ± 601.6
1998	4131	28.7 ± 5.6	3573 (86.5)	186 (4.5)	107 (2.6)	2061 (49.9)	3209.5 ± 631.4
1999	4246	28.6 ± 5.6	3613 (85.1)	157 (3.7)	110 (2.6)	2187 (51.5)	3235.4 ± 591.2
2000	4301	28.9 ± 5.5	3677 (85.5)	206 (4.8)	90 (2.1)	2232 (51.9)	3215.1 ± 610.8
2001	4418	28.9 ± 5.5	3804 (86.1)	212 (4.8)	110 (2.5)	2249 (50.9)	3216.3 ± 595.8
2002	4477	29.0 ± 5.5	3814 (85.2)	233 (5.2)	98 (2.2)	2212 (49.4)	3208.2 ± 598.2
2003	4457	29.0 ± 5.4	3900 (87.5)	200 (4.5)	85 (1.9)	2251 (50.5)	3199.9 ± 583.9
2004	4273	29.1 ± 5.5	3713 (86.9)	214 (5.0)	85 (2.0)	2226 (52.1)	3206.5 ± 581.6
2005	4232	29.3 ± 5.4	3716 (87.8)	174 (4.1)	114 (2.7)	2167 (51.2)	3192.5 ± 581.6
2006	4215	29.3 ± 5.5	3688 (87.5)	249 (5.9)	105 (2.5)	2145 (50.9)	3192.5 ± 576.2
2007	4135	29.4 ± 5.5	3684 (89.1)	265 (6.4)	87 (2.1)	2142 (51.8)	3175.1 ± 575.2
2008	4222	29.3 ± 5.4	3726 (88.3)	228 (5.4)	72 (1.7)	2124 (50.3)	3190.7 ± 562.5
2009	4171	29.7 ± 5.6	3299 (79.1)	284 (6.8)	100 (2.4)	2158 (51.7)	3179.3 ± 571.7
2010	4285	29.6 ± 5.5	3797 (88.6)	313 (7.3)	103 (2.4)	2181 (50.9)	3177.1 ± 581.7
2011	4289	29.7 ± 5.5	3779 (88.1)	279 (6.5)	116 (2.7)	2179 (50.8)	3176.5 ± 559.4
2012	4228	29.7 ± 5.6	3691 (87.3)	300 (7.1)	101 (2.4)	2173 (51.4)	3176.5 ± 559.4

Data are mean ± standard deviation or N (%).

*2 or more fetuses.

**Table 2:** Annual total births, cesarean deliveries, and major neonatal and maternal morbidities.

Year	Births	All cesareans	Elective cesareans	Non-elective cesareans	Brachial plexus injury	Neonatal encephalopathy	Placenta accreta	Hysterectomy	Blood transfusion
1993	3988	434 (10.9)	390 (9.8)	44 (1.1)	17 (0.43)	6 (0.15)	0 (0.00)	3 (0.08)	19 (0.5)
1994	4000	482 (12.1)	462 (11.6)	20 (0.5)	15 (0.38)	1 (0.03)	3 (0.08)	4 (0.10)	11 (0.3)
1995	3854	427 (11.1)	410 (10.6)	17 (0.4)	24 (0.62)	2 (0.05)	2 (0.05)	4 (0.10)	21 (0.5)
1996	3861	528 (13.7)	349 (9)	179 (4.6)	13 (0.34)	2 (0.05)	4 (0.10)	4 (0.10)	23 (0.6)
1997	4023	655 (16.3)	28 (0.7)	627 (15.6)	11 (0.27)	2 (0.05)	1 (0.02)	1 (0.02)	30 (0.7)
1998	4131	627 (15.2)	70 (1.7)	557 (13.5)	9 (0.22)	2 (0.05)	3 (0.07)	3 (0.07)	37 (0.9)
1999	4246	720 (17.0)	110 (2.6)	610 (14.4)	3 (0.07)	1 (0.02)	2 (0.05)	1 (0.02)	31 (0.7)
2000	4301	796 (18.5)	184 (4.3)	612 (14.2)	2 (0.05)	3 (0.07)	1 (0.02)	4 (0.09)	33 (0.8)
2001	4418	878 (19.9)	244 (5.5)	634 (14.4)	4 (0.09)	2 (0.05)	2 (0.05)	4 (0.09)	49 (1.1)
2002	4477	805 (18.0)	264 (5.9)	541 (12.1)	1 (0.02)	3 (0.07)	2 (0.04)	3 (0.07)	39 (0.9)
2003	4457	791 (17.7)	321 (7.2)	470 (10.5)	6 (0.13)	3 (0.07)	2 (0.04)	2 (0.04)	63 (1.4)
2004	4273	751 (17.6)	196 (4.6)	555 (13)	7 (0.16)	3 (0.07)	1 (0.02)	2 (0.05)	51 (1.2)
2005	4232	846 (20.0)	204 (4.8)	642 (15.2)	2 (0.05)	3 (0.07)	3 (0.07)	2 (0.05)	42 (1.0)
2006	4215	911 (21.6)	244 (5.8)	667 (15.8)	2 (0.05)	0 (0.00)	3 (0.07)	2 (0.05)	36 (0.9)
2007	4135	830 (20.1)	230 (5.6)	600 (14.5)	7 (0.17)	1 (0.02)	5 (0.12)	5 (0.12)	44 (1.1)
2008	4222	826 (19.6)	236 (5.6)	590 (14)	6 (0.14)	1 (0.02)	5 (0.12)	4 (0.09)	46 (1.1)
2009	4171	906 (21.7)	231 (5.5)	675 (16.2)	2 (0.05)	1 (0.02)	2 (0.05)	3 (0.07)	83 (2.0)
2010	4285	861 (20.1)	268 (6.3)	593 (13.8)	4 (0.09)	0 (0.00)	4 (0.09)	2 (0.05)	93 (2.2)
2011	4289	870 (20.3)	300 (7.0)	570 (13.3)	4 (0.09)	1 (0.02)	5 (0.12)	5 (0.12)	90 (2.1)
2012	4228	823 (19.5)	345 (8.2)	478 (11.3)	5 (0.12)	0 (0.00)	3 (0.07)	0 (0.00)	65 (1.5)
1993-2012	83806	14767 (17.6)	5086 (6.1)	9681 (11.6)	144 (0.17)	37 (0.04)	53 (0.06)	58 (0.17)	906 (1.1)

Data are N (% of Births).
